# Association of atherogenic index of plasma and triglyceride glucose-body mass index and sarcopenia in adults from 20 to 59: a cross-sectional study

**DOI:** 10.3389/fendo.2024.1437379

**Published:** 2024-08-19

**Authors:** Ruirong Pan, Tingwei Wang, Ruixue Tang, Zifan Qian

**Affiliations:** Department of Geriatrics, Affiliated Hospital of Jiangsu University, Zhenjiang, China

**Keywords:** atherogenic index of plasma, triglyceride glucose-body mass index, sarcopenia, NHANES, cross-sectional studies

## Abstract

**Background:**

The relationship between atherogenic index of plasma (AIP) and triglyceride glucose-body mass index (TyG-BMI) and sarcopenia has not been studied in the United States (US) population.

**Methods:**

This research included 4,835 people from the National Health and Nutrition Examination Survey (NHANES) conducted between 2011 and 2018. The relationship between sarcopenia and TyG-BMI, as well as the AIP index, was examined through the utilization of restricted cubic spline (RCS) analysis, subgroup analysis, and multivariate logistic regression analysis. Diagnostic value of AIP and TyG-BMI for sarcopenia was compared by receiver operating characteristic (ROC) curves.

**Results:**

In this research, 428 people with sarcopenia were identified among the 4,835 subjects that were included in the experiment. AIP and sarcopenia were positively associated with an odds ratio (OR) of 1.58 and a 95% confidence interval (CI) of (1.07, 2.34) on fully adjusted multivariate logistic regression analysis. Similarly, TyG-BMI and sarcopenia were positively associated with an OR of 8.83 and a 95% CI of (5.46, 14.26). AIP and sarcopenia had a non-linear positive connection (P-value<0.001, P-Nonlinear=0.010), while TyG-BMI and sarcopenia had a linear positive correlation (P-value<0.001, P-Nonlinear=0.064), according to RCS analysis. Subgroup analyses showed a significant interaction between TyG-BMI and sarcopenia due to gender (P = 0.023). ROC curves showed that TyG-BMI (AUC:0.738, 95% CI: 0.714 - 0.761) was more useful than AIP (AUC:0.648, 95% CI: 0.622 - 0.673) in diagnosing sarcopenia.

**Conclusion:**

In US adults aged 20–59 years, our study revealed a correlation between elevated AIP and TyG-BMI levels and heightened sarcopenia risk. Moreover, TyG-BMI has better diagnostic validity than AIP.

## 1 Introduction

The International Classification of Diseases, which was published in 2016, formally recognizes sarcopenia as a separate disorder (1). Sarcopenia is defined by decreased muscle mass, reduced strength, and progressive loss of function (2). It is estimated that 10% to 16% of older adults worldwide suffer from sarcopenia (3). Although commonly affecting the elderly, this condition can also onset in mid-life (2) and become prevalent among specific populations, including patients with cancer (4), kidney dysfunction (5), liver disease (6), and metabolic disorders (7). Compared to people without sarcopenia, those with sarcopenia have a markedly increased risk of undesirable results such as falls, disability, hospitalization, and even death (8). One of the main mechanisms of sarcopenia is the chronic low-grade inflammatory state associated with aging, commonly referred to as “inflammaging” (9). Several studies have demonstrated that inflammation initiates proteolytic metabolism and impedes anabolic reactions in skeletal muscle, resulting in muscle wasting and contributing to the development of sarcopenia (10, 11). Given the lack of effective pharmacological treatments for sarcopenia, identifying and implementing effective methods for prediction and prevention are paramount.

Atherogenic index of plasma (AIP), which was originally employed as a biomarker for atherosclerosis, is increasingly recognized as a significant forecaster of various disorders. Research indicates a robust correlation between AIP and cardiovascular risk, making it a reliable predictor of cardiovascular events and related mortality (12). Additionally, AIP has demonstrated utility in predicting hypertension, diabetes, and non-alcoholic fatty liver disease (13–15). Triglyceride glucose-body mass index (TyG-BMI) derived from the triglyceride-glucose (TyG) index, demonstrates superior predictive efficacy for insulin resistance in contrast to both the TyG index alone, as well as conventional metrics such as body mass index (BMI) and visceral fat index (16). Furthermore, a heightened risk of atherosclerotic cardiovascular disease and the onset of heart failure have been linked to higher levels of TyG-BMI (16, 17).

Prior research has linked various biomarkers to sarcopenia. For instance, Sung-Ho Ahn et al. identified a negative relationship between muscle mass and TyG in a Korean population (18). Bokun Kim proposed TyG as a potential predictor of obesity sarcopenia in the elderly (19). Additionally, Diao et al.’s meta-analysis revealed a correlation between a higher incidence of sarcopenia and greater inflammatory indices (20). It has been demonstrated that AIP can serve as a marker for assessing inflammatory status (21). The connection between AIP and TyG-BMI and sarcopenia has not yet been studied. Thus, this study uses NHANES data from 2011 to 2018 better understand the correlation between AIP and TyG-BMI and sarcopenia development in the US population.

By doing so, this research contributes to a better understanding of sarcopenia and the identification of effective predictive indicators for the disease.

## 2 Methods

### 2.1 The study’s format and the participants

**Figure 1:**
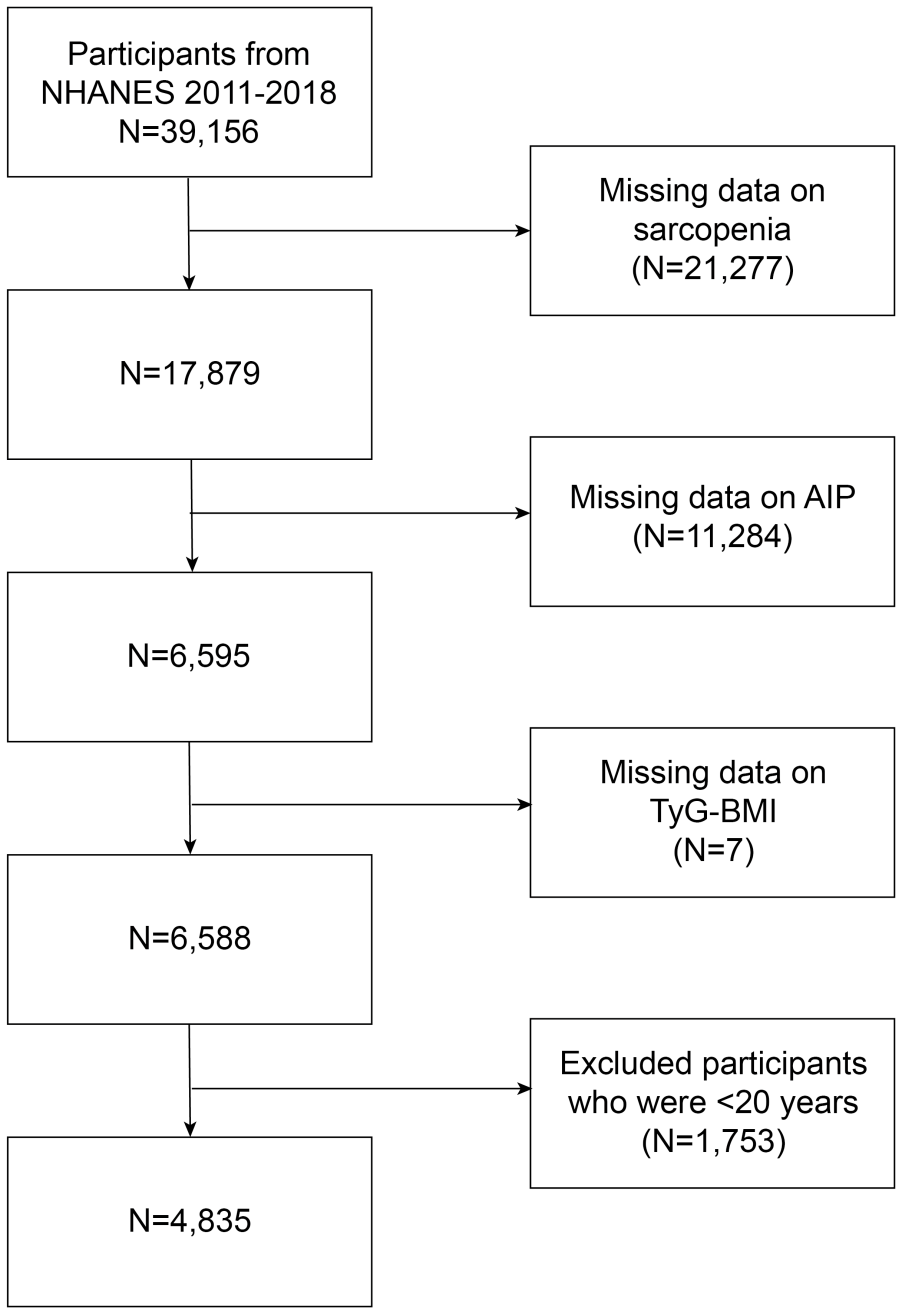
Flowchart of participants selection from NHANES 2011–2018.

The NHANES is a program for research that aims to assess the nutritional and health status of both adults and children in the United States. It consists of both physical exams and interviews and is administered by the Centers for Disease Control and Prevention. Every participant granted written permission, and the ethical review board of the National Center for Health Statistics approved all procedures [NHANES - NCHS Research Ethics Review Board Approval (cdc.gov)]. A cross-sectional examination of NHANES data from 2011 to 2018 was conducted for this study. It focused on individuals aged 20 to 59 years who underwent measurements for dual-energy X-ray absorptiometry (DXA), triglyceride, fasting glucose, and high-density lipoprotein cholesterol. Exclusions comprised individuals without DXA testing (N=21,277), AIP index (N=11,284), TyG-BMI index (N=7), and those under 20 years of age (N=1,753). Ultimately, 4,835 participants were analyzed, as depicted in **Figure 1**.

### 2.2 Definition of DIP and TyG-BMI

AIP was calculated using the logarithmic transformation of the triglycerides (TG) to high-density lipoprotein cholesterol (HDL-C) ratio with triglycerides and high-density lipoprotein cholesterol expressed in mmol/L (22). TyG-BMI was computed in this way: Weight (kg)/height squared(m^2^) equals BMI, and the TyG index is equal to Ln [1/2 fasting glucose (mg/dL) × fasting triglycerides (mg/dL)]; TyG index×BMI = TyG-BMI (23). It is important to emphasize that within the study’s framework, AIP and TyG-BMI were regarded as key exposure variables.

### 2.3 Sarcopenia

Sarcopenia, as defined by the Foundation for the National Institutes of Health (FNIH) guidelines, is described as appendicular skeletal muscle mass adjusted by body mass index (ASMBMI) of < 0.512 for female and < 0.789 for male (24). Appendicular skeletal muscle mass was determined by DXA measurements of arm and leg muscle mass. Safety precautions were implemented by excluding pregnant women and individuals weighing over 136 kg or taller than 196 cm from DXA measurements.

### 2.4 Ascertainment of covariates

Gender, age, education level, ethnicity, ratio of family income to poverty (PIR), alcohol consumption, smoking status, total calcium, potassium, sodium, total cholesterol, low density lipoprotein cholesterol (LDL-C), uric acid, red blood cell count, white blood cell count, presence of cancer, stroke status, hypertension and diabetes were all included as covariates in the statistical analysis. Stroke status was determined by the “Ever told you had a stroke” questionnaire, with a yes response indicating that the participant had a previous or current stroke. The presence of cancer was ascertained using the question, “ Ever told you had cancer or malignancy” with a positive response indicated a history of cancer for the participant. Diabetes status was ascertained through the administration of the “Doctors told you have diabetes” questionnaire, wherein positive answers indicated the presence of diabetes. Likewise, participants who responded positively on the questionnaire that “Your doctor informed you that you have high blood pressure” were categorized as having hypertension. Participants completed a questionnaire to ascertain if they had ever smoked more than 100 cigarettes. Individuals who replied in the affirmative were categorized as having previously smoked. Additionally, the questionnaire that “How often drink alcohol over past 12 months” was utilized to inquire about participants’ alcohol consumption frequency over the past year, with responses of 0 defined as never, 1–12 as sometimes, and >12 as often.

### 2.5 Statistical analyses

Mean ± standard deviation was used to express continuous variables, while frequencies (percentages) were used to express categorical variables. In order to determine the OR and corresponding 95% CI between each quartile of TyG-BMI and AIP and the prevalence of sarcopenia, multivariate logistic regression analysis was performed. The analysis comprised three logistic regression models. It was not included any covariate adjustments in Model 1, while it was included gender, age, education level, ethnicity, and PIR in Model 2. Model 3, after making complete adjustments, encompassed factors like age, gender, PIR, ethnicity, education level, total calcium, potassium, sodium, uric acid, red blood cell count, white blood cell count, total cholesterol, LDL-C, alcohol consumption, smoking status, presence of cancer, stroke status, hypertension, and diabetes. The relationship between AIP and TyG-BMI and the risk of sarcopenia development was examined through RCS analysis. Covariates including age, gender, ethnicity, education level, and PIR were considered when modifying the model. Subgroup analyses were conducted to look at any variations in the link between TyG-BMI and AIP and sarcopenia occurrence among subgroups defined by hypertension, diabetes, smoking status, alcohol consumption, gender, and age. Missing values were filled in using a random forest model. For diagnostic value analysis, ROC curves were utilized. The prognostic capacity of AIP and TyG-BMI was determined by calculating the area under the curve (AUC), as indicated by the C-statistic. And the Youden index (i.e., sensitivity + specificity-1) was utilized to find the cutoff values of AIP and TyG-BMI. The statistical studies were conducted utilizing EmpowerStats and R 4.2.2 software (https://www.empowerstats.com).

## 3 Results

### 3.1 Baseline characteristics of the population based on sarcopenia

**Table 1** displays the demographic attributes and additional variables of the study subjects, delineated by the presence of sarcopenia and categorized into quartiles based on AIP and TyG-BMI levels. In particular, participants with sarcopenia were predominantly Mexican American, with an education level of Some college or AA degree, occasional alcohol consumption, nonsmokers, and those without diabetes, hypertension, stroke and cancer. The participants with sarcopenia were older than the non-sarcopenic subjects and showed increased levels of red blood cell count, white blood cell count, serum uric acid, total cholesterol, and LDL-C, while having lower levels of serum total calcium and PIR. However, serum sodium, serum potassium levels, gender, and smoking status exhibited no significant differences between sarcopenia and non-sarcopenia participants. There was a gradual increase in the proportion of individuals with sarcopenia in both AIP and TyG-BMI quartile groupings.

**Table 1:** Baseline characteristics of participants.

Characteristic	Sarcopenia		*P*-value
	Yes, N=428	No, N=4,407	
Total Calcium, mg/dL, M ± SD	9.26 ± 0.35	9.32 ± 0.34	<0.001
Potassium, mmol/L, M ± SD	4.01 ± 0.35	3.99 ± 0.32	0.420
Sodium, mmol/L, M ± SD	139.42 ± 2.46	139.26 ± 2.14	0.177
Uric Acid, mg/dL, M ± SD	5.59 ± 1.49	5.33 ± 1.38	<0.001
Red Blood Cell, million cells/uL, M ± SD	4.85 ± 0.52	4.77 ± 0.48	0.002
Age, years, M ± SD	45 ± 11	39 ± 11	<0.001
PIR, M ± SD	2.11 ± 1.52	2.50 ± 1.60	<0.001
White Blood Cell, 1000 cells/uL, M ± SD	7.61 ± 2.25	6.76 ± 1.98	<0.001
Total Cholesterol, mg/dL, M ± SD	199 ± 41	189 ± 40	<0.001
LDL-C, mg/dL, M ± SD	121 ± 34	113 ± 34	<0.001
Alcohol Consumption, n (%)			<0.001
Never	110 (25.7%)	641 (14.5%)	
Sometimes	293 (68.5%)	3,340 (75.8%)	
Often	25 (5.8%)	426 (9.7%)	
Smoking Status, n (%)			0.918
Yes	171 (40.0%)	1,772 (40.2%)	
No	257 (60.0%)	2,635 (59.8%)	
Hypertension, n (%)			<0.001
Yes	147 (34.3%)	1,013 (23.0%)	
No	281 (65.7%)	3,394 (77.0%)	
Gender, n (%)			0.870
Male	213 (49.8%)	2,175 (49.4%)	
Female	215 (50.2%)	2,232 (50.6%)	
Race, n (%)			<0.001
Mexican American	142 (33.2%)	583 (13.2%)	
Other Hispanic	69 (16.1%)	448 (10.2%)	
Non-Hispanic White	122 (28.5%)	1,587 (36.0%)	
Non-Hispanic Black	23 (5.4%)	944 (21.4%)	
Non-Hispanic Asian	61 (14.3%)	661 (15.0%)	
Other Race	11 (2.6%)	184 (4.2%)	
Education Level, n (%)			<0.001
Less than 9th grade	82 (19.2%)	231 (5.2%)	
9-11th grade	69 (16.1%)	533 (12.1%)	
High school graduate/GED or equivalent	99 (23.1%)	936 (21.2%)	
Some college or AA degree	111 (25.9%)	1,418 (32.2%)	
College graduate or above	67 (15.7%)	1,289 (29.2%)	
Diabetes, n (%)			<0.001
Yes	65 (15.2%)	296 (6.7%)	
No	363 (84.8%)	4,111 (93.3%)	
Stroke, n (%)			0.014
Yes	12 (2.8%)	58 (1.3%)	
No	416 (97.2%)	4,349 (98.7%)	
Cancer, n (%)			0.232
Yes	20 (4.7%)	156 (3.5%)	
No	408 (95.3%)	4,251 (96.5%)	
AIP, n (%)			<0.001
Q1 [-1.25,-0.33)	46 (10.7%)	1,123 (25.5%)	
Q2 [-0.33,-0.1)	65 (15.2%)	1,174 (26.6%)	
Q3 [-0.1,0.14)	149 (34.8%)	1,067 (24.2%)	
Q4 [0.14,1.65]	168 (39.3%)	1,043 (23.7%)	
TyG-BMI, n (%)			<0.001
Q1 [115,196)	25 (5.8%)	1,184 (26.9%)	
Q2 [196,236)	58 (13.6%)	1,150 (26.1%)	
Q3 [236,283)	107 (25.0%)	1,102 (25.0%)	
Q4 [283,571]	238 (55.6%)	971 (22.0%)	

PIR, ratio of family income to poverty; LDL-C, low-density lipoproteins cholesterol; AIP, atherogenic index of plasma; TyG-BMI, triglyceride glucose-body mass index; M, mean values; SD, standard deviation.

### 3.2 Multivariate logistic regression analysis of AIP and TyG-BMI levels with sarcopenia

**Table 2** illustrates the correlation between AIP and TyG-BMI levels and sarcopenia. Individuals were stratified into four quartile groups according to AIP and TyG-BMI levels. Within the AIP quartile groups, the risk of sarcopenia was positively correlated with AIP levels, according to Model 1. Compared to the first quartile, the risk of sarcopenia increased by 241% and 293% in the third and fourth quartiles, respectively (OR=3.41, 95% CI: 2.42–4.79; OR=3.93, 95% CI: 2.81–5.51). After adjusting for gender, age, education level, ethnicity, and PIR, the risk of sarcopenia was positively correlated with AIP levels in Model 2. Compared to the first quartile, the risk of sarcopenia increased by 147% and 148% in the third and fourth quartiles, respectively (OR=2.47, 95% CI: 1.74–3.53, OR=2.48, 95% CI: 1.73–3.54). In Model 3, after controlling for gender, age, education level, ethnicity, PIR, total calcium, potassium, sodium, uric acid, red blood cell count, white blood cell count, total cholesterol, LDL-C, diabetes, hypertension, stroke, cancer, alcohol consumption, and smoking status, the risk of sarcopenia was positively correlated with AIP levels. Compared to the first quartile, the risk of sarcopenia increased by 83% and 58% in the third and fourth quartiles, respectively (OR=1.83, 95% CI: 1.26–2.68, OR=1.58, 95% CI: 1.07–2.34). In the TyG-BMI quartile groups, the risk of sarcopenia was positively correlated with TyG-BMI levels according to Model 1. Compared to the first quartile, the risk of sarcopenia rose by139%, 360% and 1061% in the second, third, and fourth quartiles, respectively (OR=2.39, 95% CI: 1.48–3.84; OR=4.60, 95% CI: 2.95–7.16; OR= 11.61, 95% CI: 7.62–17.68). In model 2, the risk of sarcopenia was positively correlated with TyG-BMI levels. Compared to the first quartile, the risk of sarcopenia increased by92%, 244%, and 934% in the second, third, and fourth quartiles, respectively (OR=1.92, 95% CI: 1.18–3.13; OR=3.44, 95% CI: 2.17–5.44; OR=10.34, 95% CI: 6.63–16.14). In model 3, the risk of sarcopenia was positively correlated with TyG-BMI levels. Compared to the first quartile, the risk of sarcopenia was increased by 82%, 204%, and 783% in the second, third, and fourth quartiles, respectively (OR=1.82, 95% CI: 1.11–2.99; OR=3.04, 95% CI: 1.89–4.89; OR= 8.83, 95% CI: 5.46–14.26).

**Table 2:** Association between AIP and TyG-BMI with sarcopenia.

	OR (95% CI), *P*-value		
	Model 1	Model 2	Model 3
AIP			
Q1 [-1.25,-0.33)	1.00(reference)	1.00(reference)	1.00(reference)
Q2 [-0.33,-0.1)	1.35 (0.92-1.99, *p*=0.126)	1.14 (0.77-1.69, p=0.523)	0.97 (0.64-1.45, *p*=0.867)
Q3 [-0.1,0.14)	3.41 (2.42-4.79, *p*<0.001)	2.47 (1.74-3.53, p<0.001)	1.83 (1.26-2.68, *p=*0.002)
Q4 [0.14,1.65]	3.93 (2.81-5.51, *p*<0.001)	2.48 (1.73-3.54, p<0.001)	1.58 (1.07-2.34, *p*=0.023)
TyG-BMI			
Q1 [115,196)	1.00(reference)	1.00(reference)	1.00(reference)
Q2 [196,236)	2.39 (1.48-3.84, *p*<0.001)	1.92 (1.18-3.13, p=0.009)	1.82 (1.11-2.99, *p*=0.017)
Q3 [236,283)	4.60 (2.95-7.16, *p*<0.001)	3.44 (2.17-5.44, p<0.001)	3.04 (1.89-4.89, *p*<0.001)
Q4 [283,571]	11.61 (7.62-17.68, *p*<0.001)	10.34 (6.63-16.14, p<0.001)	8.83 (5.46-14.26, *p*<0.001)

Model 1: crude model.

Model 2: adjusted for age, gender, race, education level and ratio of family income to poverty.

Model 3: adjusted for age, gender, race, education level, ratio of family income to poverty, total calcium, potassium, sodium, uric acid, red blood cell count, white blood cell count, total cholesterol, LDL-C, alcohol consumption, smoking status, hypertension, diabetes, cancer and stroke.

OR, odds ratio; CI, confidence interval.

### 3.3 RCS analysis investigating the relationship between AIP and TyG-BMI levels and sarcopenia

**Figure 2:**
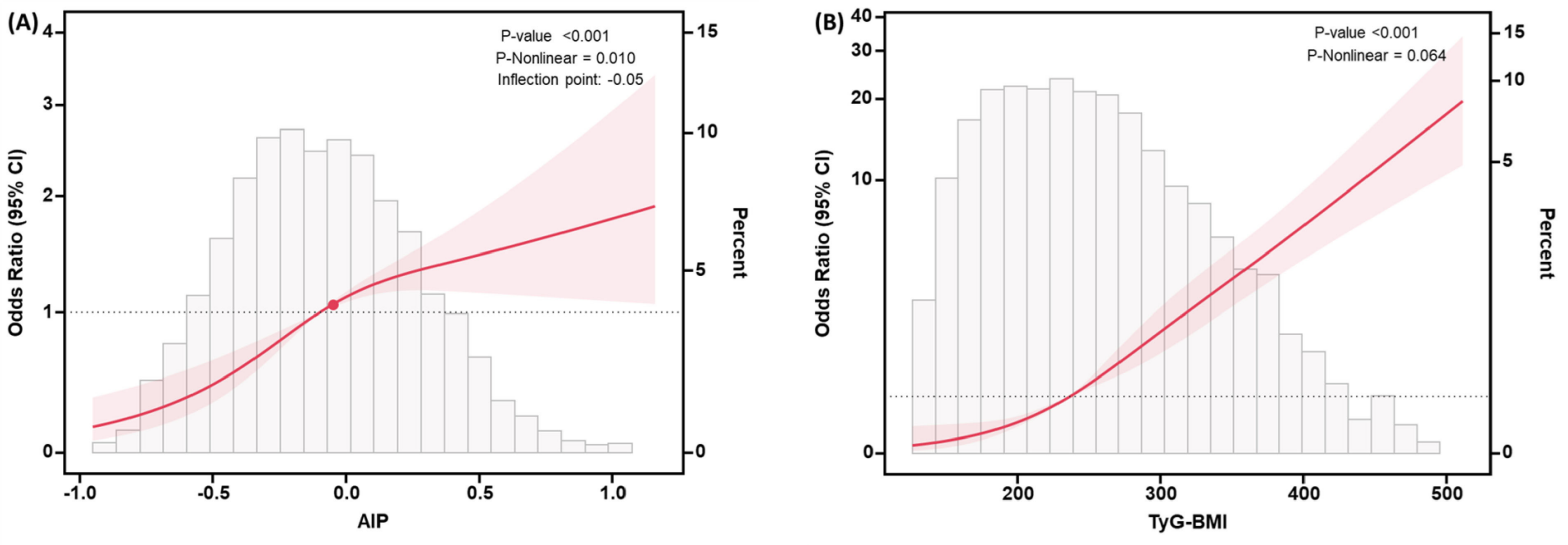
RCS curves for the association between AIP **(A)**, TyG-BMI **(B)** levels and the risk of developing sarcopenia. The model was adjusted for covariates including gender, age, ethnicity, education level, and PIR. The pink-shaded regions indicated the 95% confidence intervals and turning point was illustrated using a red dot. Abbreviations: OR, odds ratio; CI, confidence interval.

We modeled and visualized the association between AIP and TyG-BMI levels and sarcopenia using RCS analysis in **Figure 2**. After adjusting for the covariates including gender, age, ethnicity, education level, and PIR, a non-linear positive connection was found between AIP and sarcopenia (P-value<0.001, P-Nonlinear=0.010), and the OR curve was steeper when AIP<-0.05 than when AIP>-0.05, where the range of AIP was from -1.25 to 1.65. Conversely, after adjusting for the same covariates, a linear positive connection was discovered between TyG-BMI and sarcopenia (P-value<0.001, P-Nonlinear=0.064), where the range of TyG-BMI was from 114.52 to 571.42.

### 3.4 Subgroup analysis investigating the association between AIP and TyG-BMI levels and sarcopenia

**Figure 3:**
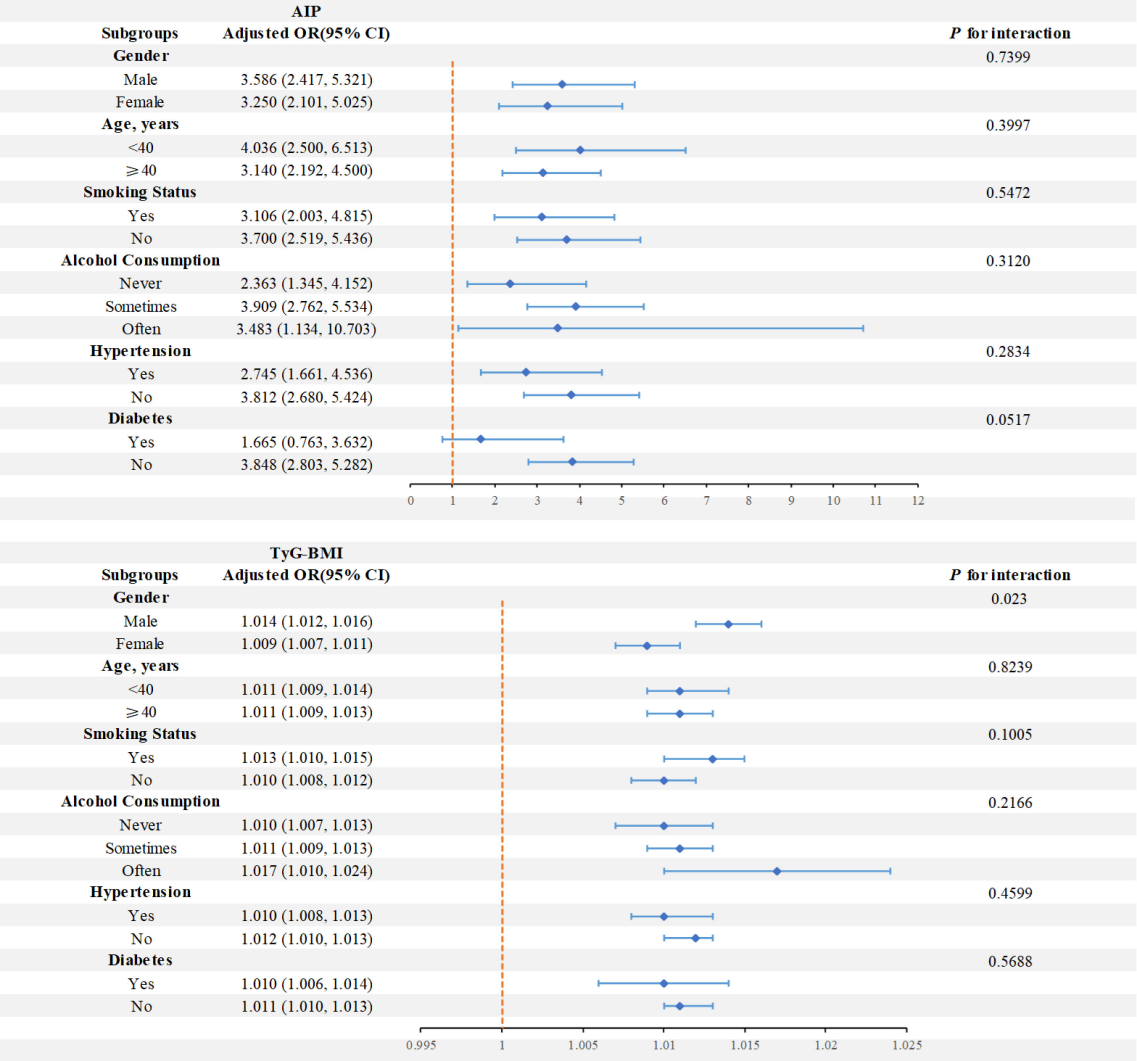
Forest plots of stratified analyses of AIP and TyG-BMI with sarcopenia. Age, gender, alcohol consumption, smoking status, and a history of diabetes and hypertension were all adjusted except the stratification variable itself.

We conducted subgroup analyses stratified by gender, age, smoking status, alcohol consumption, diabetes, and hypertension to delve deeper into the correlation between AIP and TyG-BMI levels and sarcopenia, and performed interaction tests (**Figure 3**). Concerning the correlation between AIP and sarcopenia, the findings indicated that the link between AIP and sarcopenia was not significantly affected by the variables of gender, age, alcohol consumption, smoking status, diabetes, and hypertension (P for interaction>0.05). Concerning the correlation between TyG-BMI and sarcopenia, the findings indicated that gender had a significant interaction effect on the interaction between TyG-BMI and sarcopenia (P for interaction = 0.023). The TyG-BMI range for female participants was from 114.52 to 543.15, and for male participants, it was from 115.4 to 571.42. However, this connection was not significantly impacted by age, alcohol consumption, smoking status, hypertension, or diabetes (P for interaction > 0.05).

### 3.5 ROC curves of AIP and TyG-BMI levels in diagnosing sarcopenia

**Figure 4:**
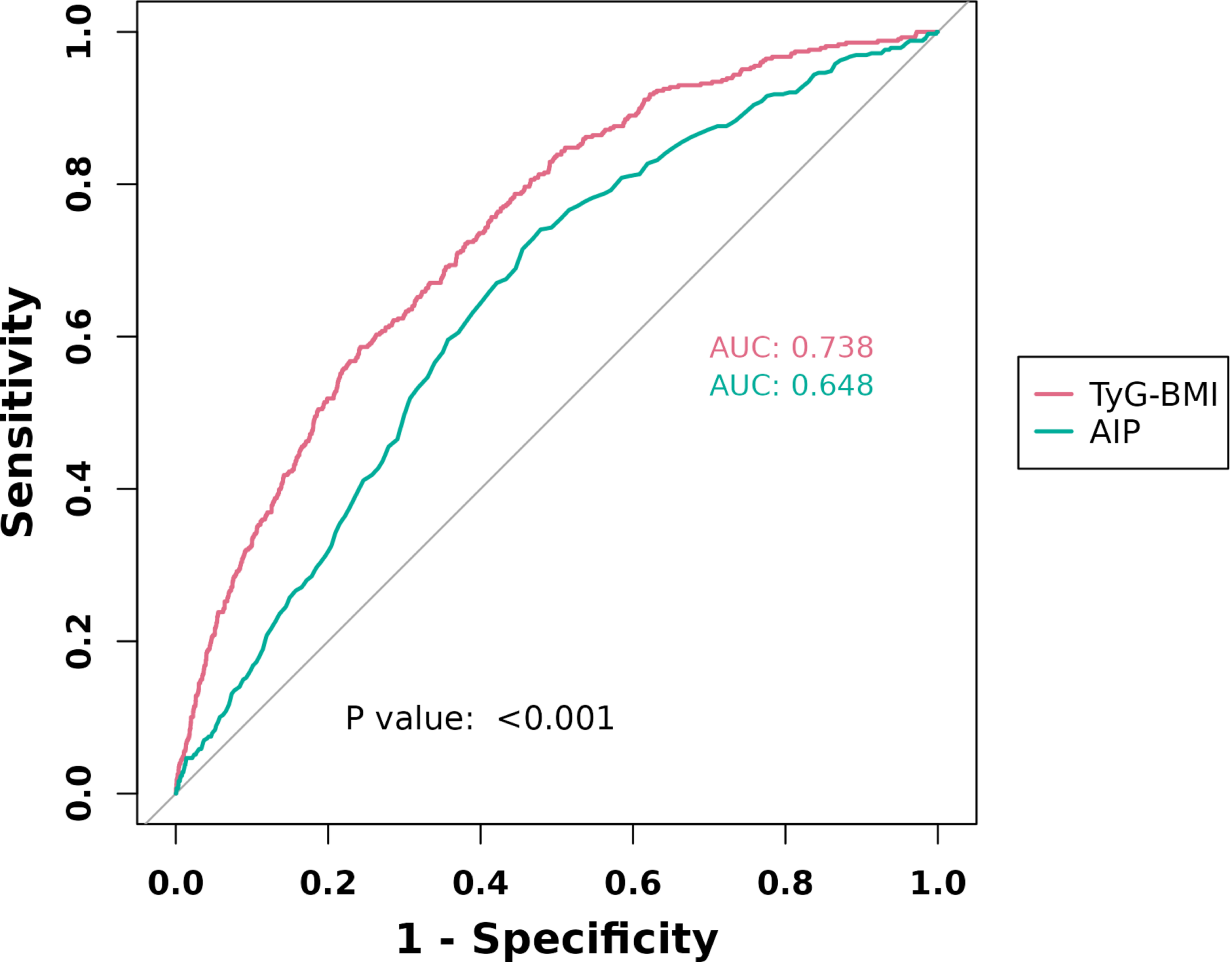
ROC curves of AIP and TyG-BMI levels in diagnosing sarcopenia.

The ROC curve (**Figure 4**) revealed that TyG-BMI exhibited superior diagnostic validity for sarcopenia (AUC: 0.738, 95% CI: 0.714 - 0.761) compared to AIP, which demonstrated lower diagnostic validity (AUC: 0.648, 95% CI: 0.622 - 0.673). The cutoff value of AIP was -0.1050, and that of TyG-BMI was 277.5750. When AIP was -0.1050, the sensitivity for diagnosing sarcopenia was 0.741, and the specificity was 0.521. When TyG-BMI was 277.5750, the sensitivity for diagnosing sarcopenia was 0.5864, and the specificity was 0.7579.

## 4 Discussion

This cross-sectional study utilized data from the 2011–2018 NHANES surveys, concentrating on individuals aged 20 to 59 years, to examine the relationship between TyG-BMI and AIP and sarcopenia. After controlling for pertinent confounding factors, we observed that increased levels of AIP and TyG-BMI showed significant associations with a heightened risk of sarcopenia in our analysis of 4,835 subjects. We detected a nonlinear positive link between AIP and sarcopenia after adjusting for covariates, alongside a linear positive relationship between TyG-BMI and sarcopenia. Furthermore, we observed that the link between AIP levels and heightened sarcopenia risk did not significantly differ across subgroups based on gender, age, hypertension, diabetes, smoking status, or alcohol consumption. However, the link between increased TyG-BMI levels and elevated sarcopenia risk was notably more prominent within the male subgroup. Furthermore, compared to AIP, TyG-BMI shown better diagnostic validity for sarcopenia.

To our knowledge, this study represents the first attempt to explore the correlation between AIP and TyG-BMI and sarcopenia, as well as to compare their diagnostic efficacy for sarcopenia. Numerous pathophysiological mechanisms, such as hormone fluctuations, denervation, and mitochondrial dysfunction, are thought to be involved with sarcopenia (25). Additionally, inflammation is a major factor in sarcopenia. Increased levels of inflammatory cytokines associated with aging and disease processes can exacerbate age-related anorexia and cause a reduction in food intake, which in turn can lead to muscle loss (26). Sarcopenia may ensue from this cascade, and sarcopenia may have negative health effects like functional decline, falls, weakness, and mortality from the loss of lean body mass (25, 27). Although sarcopenia is usually linked to advanced aging, it is now known to start earlier than 60, with a decrease in muscular strength starting at roughly 40 (2, 27). Apart from the elderly population, individuals who are underweight, women, and those with other chronic conditions are also at a higher risk of developing sarcopenia (28). Scholars that have delved into biomarkers associated with sarcopenia have become more numerous in recent years. Many studies have underscored the robust relationship between sarcopenia and various biomarkers, such as the serum creatinine/cystatin C ratio (29), γ-glutamyltransferase (30), HOMA-IR index (31), etc., elucidating the intricate mechanisms underlying the condition. Prior research has demonstrated a heightened risk of sarcopenia in individuals with diabetes and its complications, along with an association between sarcopenia and heart disease (32). Our study reaffirmed these connections between the musculoskeletal, endocrine, and cardiovascular systems using two biomarkers, AIP and TyG-BMI.

AIP was first described by Dobiásová et al. in 2000 (33). It is regarded as a promising cardiovascular disease(CVD) prediction biomarker (34). Although there have been no studies specifically addressing the correlation between AIP and sarcopenia, Liu X et al. (35) have shown that there is a unidirectional causal relationship between sarcopenia and cardiovascular disease, and the loss of muscle mass and strength has a significant causal role in promoting the occurrence and development of CVD. Gao K et al. (36) also found that among Chinese individuals in their middle and later years, sarcopenia and potential sarcopenia were linked to an increased risk of cardiovascular disease. Our investigation turned up a substantial positive connection between AIP and sarcopenia in the American community. Previous investigations have looked into the correlation between AIP and various diseases using a range of epidemiological techniques. Kim, S.H. et al. (37) revealed an established correlation between AIP and the risk of CVD after adjusting for traditional risk factors, suggesting that AIP may be utilized to identify patients at high risk for cardiovascular events. AIP outperformed other lipid components in predicting diabetes risk, according to a meta-analysis of 15 case-control studies (38). Additionally, several researches suggest that AIP may serve as a potential marker of inflammation and impaired lipid metabolism (39, 40).

TyG-BMI serves as an indicator for evaluating insulin resistance, with Er LK et al. (41) demonstrating its superiority over other insulin resistance scores, including HOMA-IR, for identifying insulin resistance. Additionally, Lim J et al. (42) reported that TyG-BMI outperformed TyG and triglyceride glucose-waist circumference (TyG-WC) in predicting insulin resistance. Sarcopenia and TyG-BMI levels were found to be significantly positively correlated by our investigation. While the association between TyG-BMI levels and sarcopenia has not been specifically studied, previous research in various populations has linked TyG levels with sarcopenia. A study conducted in a Korean population (43) revealed that individuals with skeletal muscle mass relative to the lowest quartile of body weight exhibited a twofold increased risk of type 2 diabetes development compared to those in the highest quartile. Chen Y. et al. (44) found a negative correlation between the TyG index and the incidence of sarcopenia among elderly Chinese individuals. However, this association lost significance after adjustment for body mass index. In a non-diabetic cohort receiving maintenance hemodialysis, Chen R et al. (45) identified a significant correlation between the risk of sarcopenia and the TyG index. Lower skeletal muscle mass was revealed to be independently correlated with insulin resistance in older adults by Lee SW et al. (46). These findings underscore a plausible association between diabetes and sarcopenia.

To date, no study has delineated the mechanistic underpinnings of the association between AIP and TyG-BMI and sarcopenia. We posit that inflammation could serve as a pivotal mechanism in this relationship. One notable risk factor for diabetes and cardiovascular disease is chronic inflammation (47). Atherosclerosis initiates with endothelial damage, leading to the accumulation and oxidation of cholesterol-containing LDL particles in the arterial wall, triggering an unresolvable inflammatory response (48), which itself generates, activates, and sustains antigens, perpetuating the inflammatory response (47). On the other hand, chronic, low-grade systemic inflammation correlates with type 2 diabetes, exerting detrimental effects on glucose and muscle homeostasis (49–51). Elevated levels of inflammatory markers such C-reactive protein (CRP), interleukin-6 (IL-6), and tumor necrosis factor-α (TNF-α) are commonly observed in patients with type 2 diabetes (52, 53). Furthermore, inflammatory conditions impair vascular reactivity and insulin delivery, fostering insulin resistance (54). Additionally, inflammation indirectly diminishes the concentrations of growth hormone and insulin-like growth factor-1 (IGF-1), thereby adversely impacting skeletal muscle (55, 56) and fostering sarcopenia development. TNF-α impedes muscle protein synthesis through modulation of the PI3K/Akt/mTOR signaling pathway and fosters muscle atrophy via upregulating the expression of various muscle growth inhibitory factors, including atrogin1, nuclear factor κB (NF-κB), and myostatin (57, 58). Moreover, sarcopenia is regarded as an age-related condition, and increasing inflammatory markers and associated variables are frequently observed with aging (59). Age-related increases in circulation levels of inflammatory markers, such as TNF-α, CRP, interleukin-1β (IL-1β), and IL-6, are directly linked to a decline in muscle mass and strength (60–62). Therefore, we supposed that inflammation is one of the important mechanisms as a mediator of AIP and TyG-BMI prediction of sarcopenia.

This study offers several advantages. Firstly, it serves as a significant reference for further delineating and predicting the emergence of sarcopenia within the American population. Secondly, by using a nationally representative sample and a standardized experimental testing technique, along with data from NHANES, the study’s errors were significantly reduced. This approach bolstered the reliability of the experimental outcomes and guaranteed the study’s representativeness. Nonetheless, the study bears limitations. Primarily, due to its cross-sectional nature, establishing a causal link between elevated levels of AIP and TyG-BMI and heightened sarcopenia risk was unattainable. Furthermore, only individuals aged 8–59 underwent dual-energy X-rays for sarcopenia confirmation, neglecting those aged 60 and above. These limitations suggest the necessity for cohort studies in the future to determine a causal association between the two indices and sarcopenia, as well as additional studies targeting sarcopenia in the elderly population.

In summary, our research showed a correlation between higher levels of AIP and TyG-BMI and heightened sarcopenia risk in US adults aged 20–59 years. Moreover, TyG-BMI has better diagnostic validity than AIP.

## Data Availability

The datasets presented in this study can be found in online repositories. The names of the repository/repositories and accession number(s) can be found below: https://www.cdc.gov/nchs/nhanes/index.htm.
